# Dynamics of alternative polyadenylation in single root cells of *Arabidopsis thaliana*

**DOI:** 10.3389/fpls.2024.1437118

**Published:** 2024-09-20

**Authors:** Xingyu Bi, Sheng Zhu, Fei Liu, Xiaohui Wu

**Affiliations:** ^1^ Cancer Institute, Suzhou Medical College, Soochow University, Suzhou, China; ^2^ Operational Technology Research and Evaluation Center, China Nuclear Power Operation Technology Corporation, Ltd, Wuhan, China; ^3^ Jiangsu Key Laboratory of Infection and Immunity, Soochow University, Suzhou, China

**Keywords:** alternative polyadenylation, single-cell RNA-seq, root development, RNA processing, bioinformatics and computational biology

## Abstract

**Introduction:**

Single-cell RNA-seq (scRNA-seq) technologies have been widely used to reveal the diversity and complexity of cells, and pioneering studies on scRNA-seq in plants began to emerge since 2019. However, existing studies on plants utilized scRNA-seq focused only on the gene expression regulation. As an essential post-transcriptional mechanism for regulating gene expression, alternative polyadenylation (APA) generates diverse mRNA isoforms with distinct 3’ ends through the selective use of different polyadenylation sites in a gene. APA plays important roles in regulating multiple developmental processes in plants, such as flowering time and stress response.

**Methods:**

In this study, we developed a pipeline to identify and integrate APA sites from different scRNA-seq data and analyze APA dynamics in single cells. First, high-confidence poly(A) sites in single root cells were identified and quantified. Second, three kinds of APA markers were identified for exploring APA dynamics in single cells, including differentially expressed poly(A) sites based on APA site expression, APA markers based on APA usages, and APA switching genes based on 3′ UTR (untranslated region) length change. Moreover, cell type annotations of single root cells were refined by integrating both the APA information and the gene expression profile.

**Results:**

We comprehensively compiled a single-cell APA atlas from five scRNA-seq studies, covering over 150,000 cells spanning four major tissue branches, twelve cell types, and three developmental stages. Moreover, we quantified the dynamic APA usages in single cells and identified APA markers across tissues and cell types. Further, we integrated complementary information of gene expression and APA profiles to annotate cell types and reveal subtle differences between cell types.

**Discussion:**

This study reveals that APA provides an additional layer of information for determining cell identity and provides a landscape of APA dynamics during Arabidopsis root development.

## 1 Introduction

Recent advances in single-cell RNA-seq (scRNA-seq) have opened an unprecedented opportunity to profile transcriptome-wide cell-to-cell variability in any given organism. In particular, scRNA-seq has revolutionized studies in animals to facilitate the capture of cellular heterogeneity in gene expression profiles, discovery of new cell types, and reconstruction of developmental trajectories (Saliba et al., 2014; Kolodziejczyk et al., 2015; Wang and Navin, 2015). In contrast to the massive studies on animal tissues, the application of scRNA-seq in plants did not begin until 2019, which mainly because that the presence of plant cell walls hinders the isolation of individual cells. Thus far, most scRNA-seq studies in plants focused on the well characterized Arabidopsis root system (Denyer et al., 2019; Jean-Baptiste et al., 2019; Ryu et al., 2019; Shulse et al., 2019; Zhang et al., 2019; Shahan et al., 2022). Generally, these abundant studies utilized routine scRNA-seq analytical pipelines, such as Seurat (Butler et al., 2018; Stuart et al., 2019) and Scanpy (Wolf et al., 2018), to analyze the single-cell gene abundance data, which define major or minor cell types in Arabidopsis root (*e.g.*, epidermis, endodermis, cortex, stele, and root cap) and reveal developmental trajectories of major cell types.

Current studies aiming to dissert cell types from scRNA-seq relied mostly on the gene-cell abundance matrix to perform gene-level analysis. More recently, one interesting line of research, leveraging the isoform resolution of scRNA-seq data, revealed prevalent cell-to-cell heterogeneity in isoform expression and found cell-type-specific isoforms (Shalek et al., 2013; Marinov et al., 2014; Velten et al., 2015; Song et al., 2017; Arzalluz-Luque and Conesa, 2018). Previously, a number of studies based on 3′ end sequencing or bulk RNA-seq have highlighted the importance of alternative polyadenylation (APA) in regulating gene expression and affecting mRNA stability, translation and localization (Tian and Manley, 2017; Gruber and Zavolan, 2019). APA is a crucial regulatory mechanism generating diverse mRNA isoforms with distinct 3′ ends, which contributes to transcriptome diversity. Most APA sites located within the terminal exon downstream of the stop codon, resulting in transcript isoforms with distinct 3′ UTRs (untranslated regions). A small portion of APA sites are located within internal introns/exons, which may produce mRNA isoforms encoding distinct proteins. APA is prevalent and conserved across all eukaryotes and up to 70% of genes in plants possess APA sites (Wu et al., 2011; Thomas et al., 2012; Fu et al., 2016; Zhou et al., 2019; Wang et al., 2023). APA is also highly modulated in a tissue-specific manner and is involved in proliferation, development, differentiation, and disease (Tian and Manley, 2017; Gruber and Zavolan, 2019). In plants, APA has been indicated important in controlling flowering time (Xing et al., 2008), responding oxidative stress (Thomas et al., 2012) and high-salt environments (Wang et al., 2023), and regulating agriculturally important traits (Fu et al., 2016). Given the importance of APA, computational approaches have been proposed to derive additional information on APA isoforms from standard scRNA-seq data without redesigning sequencing experiments (Ye et al., 2023). For example, using scRNA-seq data from 10X Chromium, our group investigated cell-type-specific APA dynamics in acute myeloid leukemia (Ye et al., 2019a) and released the scDAPA tool for analyzing APA dynamics across different cell types (Ye et al., 2019b). By pooling cells of the same cell type from full-length scRNA-seq to mimic the analysis in bulk RNA-seq, Kim et al. found that APA usages combined with gene expression contribute to separating tumor and non-tumor cells (Kim et al., 2019). Lately, a few computational tools are emerging for identifying APA sites in individual cells from scRNA-seq data [reviewed in (Ye et al., 2023)], such as scAPA (Shulman and Elkon, 2019), Sierra (Patrick et al., 2020), and scAPAtrap (Wu et al., 2021). Based on a similar strategy of peak identification, these tools identify and quantify single-cell APA sites at the whole genome level from 3′ tag-based scRNA-seq, such as 10X Chromium, CEL-seq, and Drop-seq. The single-cell profile of APA isoform usages provides an additional layer of gene expression regulation, which contributes greatly to the discovery and annotation of new cell types. For example, the APA profile, independent of gene expression, has been shown to help discern cell identities in different stages during mouse sperm cell differentiation (Wu et al., 2021).

Arabidopsis root comprises the complex structure with diverse but relatively small number of cell types, and numerous tissue/cell-type marker genes have been defined by abundant genomic studies (Birnbaum et al., 2005; Bruex et al., 2012; Efroni et al., 2015; Li et al., 2016), which makes it an ideal plant tissue for the application of scRNA-seq. Recently, several landmark studies using scRNA-seq on Arabidopsis roots have revealed major cell types such as pericycle cells as well as very small cell populations such as quiescent center (QC) cells (Denyer et al., 2019; Jean-Baptiste et al., 2019; Ryu et al., 2019; Shulse et al., 2019; Zhang et al., 2019; Shahan et al., 2022). However, even in this well studied system, there are still cell clusters cannot be annotated due to the lack of marker genes. As a highly tractable and well characterized system, Arabidopsis root is also an ideal tissue for the study of cell-to-cell heterogeneity of APA and cell-type-specific APA in plants, especially with the availability of a compendium of scRNA-seq datasets from many pioneering studies. Recent studies based on standard scRNA-seq have revealed cell-type-specific APA regulation and discovered cell subpopulations invisible to conventional gene expression analysis (Gao et al., 2021; Wu et al., 2021). Therefore there is a great potential to detect more robust marker genes for dissecting cell types in roots based on complementary information from both layers of APA isoforms and genes derived from the same scRNA-seq experiments.

In this study, we comprehensively compiled a single-cell APA atlas from five scRNA-seq studies, covering over 150,000 cells spanning four major tissue branches, twelve cell types, and three developmental stages. High-confidence poly(A) sites in single root cells were identified and quantified. Moreover, three kinds of APA markers were identified for exploring the dynamic APA usages in single cells, including differentially expressed poly(A) sites based on APA site expression, APA markers based on the APA ratios, and APA switching genes based on 3′ UTR length. By integrating both the APA information and the gene expression profile, cell type annotations of single root cells were refined. This study reveals that APA provides an additional layer of information for determining cell identity and provides a landscape of APA dynamics during Arabidopsis root development.

## 2 Materials and methods

### 2.1 Datasets

A total of 28 scRNA-seq datasets of wild-type Arabidopsis root tissue from five studies were collected for this study (**Supplementary Table S1**). These data cover 150,697 single cells sequenced by 10X Chromium or Drop-seq, including 1,076 cells from roots of 7-day-old seedlings (Jean-Baptiste et al., 2019), 7,522 cells from 5-day-old root tips (Ryu et al., 2019), 31,578 cells from 5- and 7-day-old whole roots (Shulse et al., 2019), 7,695 cells from root tip tissue 10 days after growth (Zhang et al., 2019), and 102,826 cells from 5- and 7-day-old primary root tips (Shahan et al., 2022).

### 2.2 Annotation of cell types in Arabidopsis roots

We used the single-cell gene expression profiles to annotate cell types. The gene-cell expression matrices were obtained from the original studies. For each matrix, we retained cells containing at least 200 expressed genes, and genes expressed in at least three cells. We also excluded mitochondrial genes, chloroplast genes, or genes affected by protoplasts. The gene expression profile of each matrix was normalized using *SCTransform* function in Seurat. To construct a unified expression profile at single-cell resolution, gene-cell expression matrices from individual studies were combined by gene IDs. Then we retained genes expressed in all matrices and kept cells containing at least 500 expressed genes, and genes expressed in at least five cells. Finally, a matrix with 12,191 genes in 128,549 cells was obtained. Then batch effects removed using *IntegratedData* function in Seurat (Butler et al., 2018). Similar to the previous study (Shahan et al., 2022), we integrated three strategies to annotate the cell type of each cell to improve the reliability. First, we collected published microarray data and RNA-seq data from Arabidopsis roots to compile the reference gene expression profile (Brady et al., 2007; Li et al., 2016). Pearson’s correlation coefficient was then calculated between the expression profile of each cell and the reference profile. Second, we downloaded gene expression profiles of Arabidopsis root tissue cells from the Arabidopsis Genus Gene Expression Database (AREX LITE) and then used the Index of Cell Identity (ICI) (Birnbaum and Kussell, 2011; Efroni et al., 2015) to determine cell identity. Third, we clustered cells based on gene expression profiles and identified cluster-specific genes based on differential analysis. Then we referred to known cell type-specific marker genes to determine cell types. Each cell would be assigned one cell type by each strategy. Cells that were assigned the same cell type in at least two strategies were defined as labeled cells, and remaining cells were defined as unlabeled cells. Next, for labeled cells, the average expression levels of genes in different cell types were calculated, and the average expression levels of the top 200 highly variable genes in different cell types were selected as the reference expression profile. Pearson’s correlation coefficient was calculated between the expression profile of each unlabeled cell and the reference profiles of labeled cells, and the category label of the cell group with the highest correlation coefficient was used as the initial identity of the unlabeled cell. Finally, we annotated eleven sub-cell types, yet not covering the stem cell niche. Further, we combined the cell category labels (four in total) obtained by different strategies. Next, we filtered out the quiescent center cells not assigned with quiescent center in at least two from the four cell category labels and re-annotated these quiescent center cells as stem cell niche.

### 2.3 Identification of poly(A) sites from scRNA-seq

We used scAPAtrap (Wu et al., 2021) to identify poly(A) sites at the single-cell level from scRNA-seq data. First, Cell Ranger (Zheng et al., 2017) was used for sequence alignment for 10X data. For data from other sequencing protocols, such as Drop-seq, we first extracted the cell barcodes and unique molecular identifiers (UMIs) from the read 1 file using UMI-tools (Smith et al., 2017), and appended the read identifiers to the read 2 file to generate a new file. The new read 2 file was aligned to the reference genome by STAR to generate a BAM (Binary Alignment and Map) file (Dobin et al., 2013). The TAIR9 reference genome was used for alignment, and the Arabidopsis genome BSgenome object (“BSgenome.Athaliana.TAIR.TAIR9”) with TAIR10 gene annotation file was used in scAPAtrap for poly(A) site identification and annotation. Genome-wide poly(A) sites were identified from the BAM files using scAPAtrap (Wu et al., 2021), which pinpointed the location of each poly(A) site and quantified the expression of the poly(A) site in each cell. Next, the *annotatePAC* function of movAPA (Ye et al., 2021) was used to annotate poly(A) sites with different genomic regions. Similar to previous studies (Wu et al., 2011; Zhu et al., 2020; Wu et al., 2021), the annotated 3′ UTR regions were extended by 1000 bp, using the *ext3UTRPACds* function of movAPA, to include more potential 3′ UTR poly(A) sites in downstream regions. Only poly(A) sites located in 3′ UTR or extended 3′ UTR regions of protein-coding genes were retained for further analysis. Finally, for each scRNA-seq dataset, we obtained a poly(A) site expression matrix, with each row denoting a poly(A) site and each column a cell. The annotation information of each poly(A) site, including the chromosome location, the peak range, gene, and strand, was also recorded.

### 2.4 Integration of poly(A) sites from multiple sources

Unlike genes in a gene expression matrix, which are annotated by gene names, each poly(A) site in a poly(A) site matrix is represented by a peak region. Consequently, the peak intervals assigned to the same poly(A) site may not fully overlap across different samples. To compile a map of poly(A) sites at the whole-genome level, we used a snowball like approach to merge poly(A) sites from different sources. Briefly, poly(A) sites of all datasets were sorted according to their peak starting positions. Then poly(A) sites (peaks) with intersecting peak intervals were combined into the same site and assigned the same ID. The most distal peak end was set as the coordinate of the combined poly(A) site. This process was repeated until all poly(A) sites were processed. After re-annotating peak ranges of poly(A) sites from all sources, the expression level (read count) of each poly(A) site in each cell of each dataset was recalculated. To further eliminate batch effects, poly(A) sites from different datasets were integrated using the *RunHarmony* function in harmony (Korsunsky et al., 2019). Finally, an integrated poly(A) site expression matrix from different samples was obtained.

### 2.5 Identification of APA markers with differential APA usages

Three kinds of APA markers were identified, including differentially expressed poly(A) sites (DEPAs) based on APA site expression, APA markers based on APA usages, and APA switching genes based on 3′ UTR length change.

Based on the poly(A) site expression matrix, DEPAs for each cell type were identified using the Seurat package (Butler et al., 2018; Stuart et al., 2019). For each poly(A) site, the expression profile in a given cell group was compared with expression profile of cells in the remaining cell groups. The *P* values were adjusted for multiple hypothesis testing using the Bonferroni method. We used *FindAllMarkers* function in Seurat (parameters: test.use = wilcox, min.pct = 0.25, logfc.threshold = 0.25) to initially identify DEPAs. Resulting DEPAs with expression ratio greater than 20% cells of a given cell group and less than 20% in other cell groups were retained.

To quantify APA dynamics that reflect the change of 3′ UTR length of genes, first we obtained genes with at least two poly(A) sites in 3′ UTR (called 3′ UTR-APA genes). Further, the percentage of the proximal poly(A) site usage index (PPUI) was calculated (Equation 1). The PPUI for gene *i* in cell *s* was calculated as the ratio of the read counts for proximal poly(A) site *p* ( $E_{p,i}$ ) to the total read counts for all 3′ UTR poly(A) sites on that gene.


(1) $$PPUI_{s,i}=\frac{E_{p,i}}{\sum E_{i}}$$

The PPUI score ranges between 0 and 1, which represents the relative 3′ UTR length of a gene in a cell. A higher PPUI indicates increased use of the proximal site, *i.e.* shortening of the 3′ UTR, whereas a lower PPUI indicates lengthening of the 3′ UTR.

Next, we determined APA-switching genes that exhibit significant 3′ UTR length change among cell types. Genes with differential APA usages (called APA markers) were identified by test the difference of APA ratios between two cell groups using Wilcoxon rank sum test. To further determine the direction of APA switching (*i.e.*, shortening or lengthening), we first calculated the relative 3′ UTR length for each cell group (Equation 2). Briefly, the average expression of the poly(A) site *i* in a given cell *s* was calculated ( $E_{s,i}$ ), and the distance of poly(A) site *i* to the nearest stop codon (*i.e.*, 3′ UTR length) was obtained ( $L_{i}$ ). Then the relative 3′ UTR length of a gene with *n* poly(A) sites in cell *s* was calculated as the 3′ UTR length weighted by the expression level.


(2) $$L_{s}=\frac{\sum_{i=1}^{n}E_{s,i}*L_{i}}{\sum_{i=1}^{n}E_{s,i}}$$

Finally, the difference of relative 3′ UTR length of each gene between the two cell groups was further tested by Wilcoxon rank sum test.

### 2.6 Refining cell type annotations by combining gene and APA profiles

Cells remained unannotated after applying above three strategies on the gene-cell expression matrix were considered as unlabeled cells. Unlabeled cells present in both the integrated gene expression matrix and APA matrix were retained for further cell type annotation. We used three kinds of gene sets, including DEPA gene sets, APA marker sets, and highly variable gene (HVG) sets, for cell type annotation. First, we used the *GeneSetCollection* function in the GSEABase package to construct sets of DEPAs targeting the twelve cell types in Arabidopsis roots. Next, the activity degree of different DEPA sets in each cell was calculated using the *AUCell_calcAUC* function in AUCell (Aibar et al., 2017). The area under curve (AUC) was used to calculate the proportion of poly(A) sites from different DEPA sets that were highly expressed in each cell. The cell type corresponding to the DEPA set with a higher activity degree for each cell was considered as the cell identity. Similarly, Unlabeled cells were also re-annotated using the APA marker sets. Moreover, unlabeled cells were annotated based on the gene expression profile. Firstly, top 200 HVGs for each cell type were selected based on the gene-cell expression matrix of labeled cells, and the average expression profile of HVGs in each cell type was considered as cell-type specific reference profile. Then the Pearson’s correlation coefficient between the expression profile of each unlabeled cell and the reference profile of each cell type was calculated. Finally, the cell label with the highest correlation coefficient was considered as the identity of the unlabeled cell. By combining the annotation results based on gene expression, DEPAs, and APA markers, we used the cell type with the highest frequency of occurrence among the three annotation results as the identity of the cell. If the three annotation results are different from each other, then the annotation result based on DEPAs was used.

## 3 Results

### 3.1 A pipeline to identify APA sites and analyze APA dynamics in single root cells of Arabidopsis

**Figure 1:**
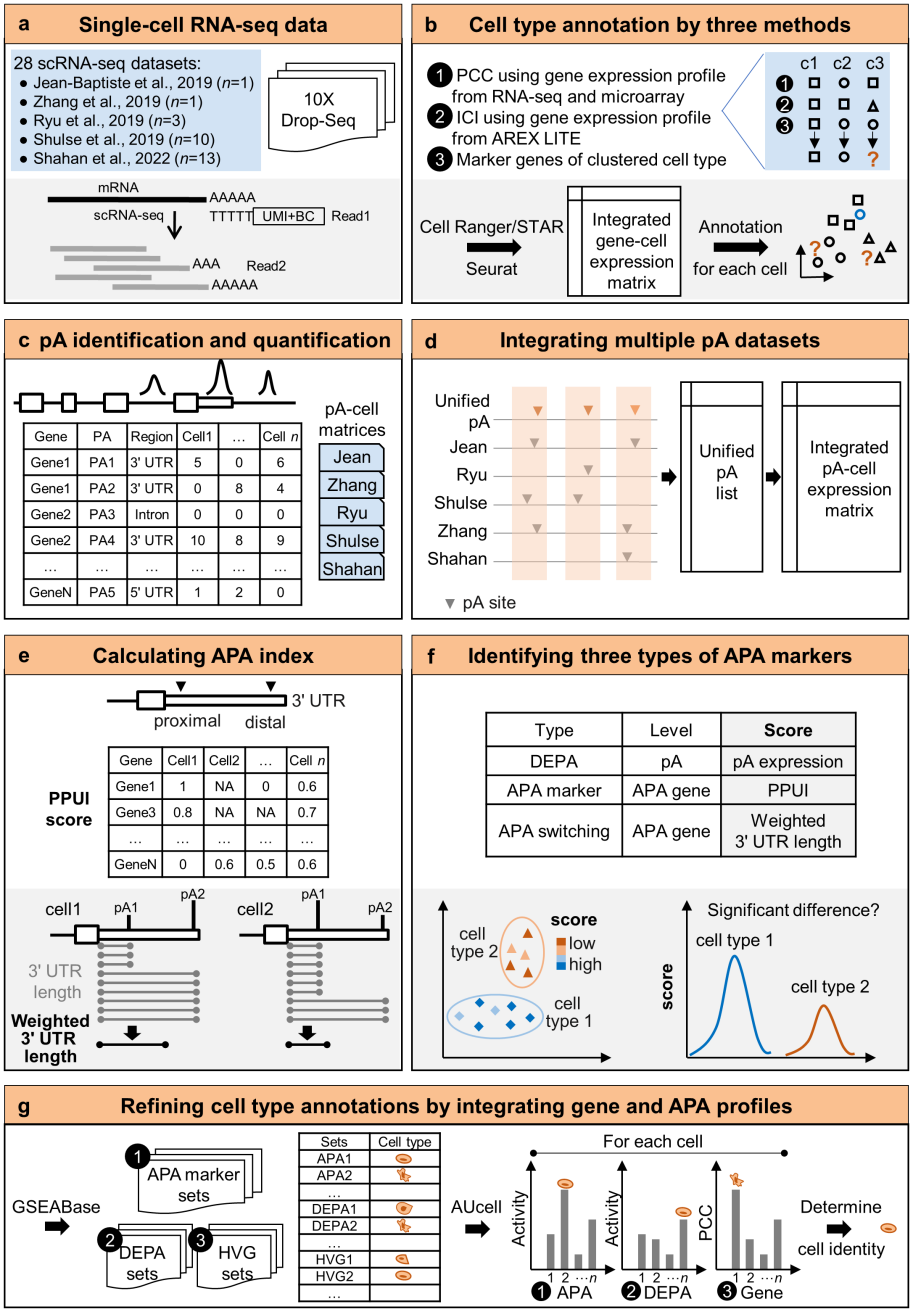
A pipeline to integrate APA sites and analyze APA dynamics in single cells. **(A)** Single-cell RNA-seq datasets collected from five studies. **(B)** Cell type annotation by three methods using gene expression profiles. **(C)** Single-cell poly(A) site identification and quantification. **(D)** Integration of multiple poly(A) site datasets. **(E)** Calculation of two APA metrics based on the integrated poly(A) site matrix. **(F)** Identification of three types of genes with APA dynamics **(G)** Refining cell type annotations by integrating gene and APA profiles. pA, poly(A) site; DEPA, differentially expressed poly(A) site; PPUI, proximal poly(A) site usage index. PCC, Pearson’s correlation coefficient; ICI, index of cell identity.

In this study, we developed a pipeline to identify and integrate APA sites from different scRNA-seq data and analyze APA dynamics in single cells. A total of 28 scRNA-seq datasets of Arabidopsis root tissue sequenced by 3′ tag based scRNA-seq were collected from five studies (**Figure 1A**). Then gene-cell expression profiles of the five studies were integrated. Three strategies based on gene expression profiles from bulk data and cell type-specific marker genes were proposed to annotate the cell type of each cell (**Figure 1B**). Cells that were not assigned the same cell type in at least two strategies were defined as unlabeled cells. Next, poly(A) sites at the single-cell level from each scRNA-seq dataset were identified and quantified, and 28 single-cell poly(A) site matrices were obtained (**Figure 1C**). To integrate poly(A) sites from multiple studies, a unified poly(A) site list was compiled and then the expression levels of poly(A) sites in each cell of each dataset were re-calculated based on the unified list (**Figure 1D**). Further, two APA metrics, including the percentage of the proximal poly(A) site usage index (PPUI) and the weighted 3′ UTR length, were used to measure APA dynamics of each gene in each cell (**Figure 1E**). Next, three kinds of APA markers were identified by testing the difference of score (*i.e.*, APA site expression, PPUI, and weighted 3′ UTR length) between two cell groups (**Figure 1F**). Finally, by combining information of gene expression and APA profiles, cell type annotations of unlabeled cells could be refined (**Figure 1G**).

### 3.2 Integration of gene expression profiles from multiple scRNA-seq studies reveals major root cell types

**Figure 2:**
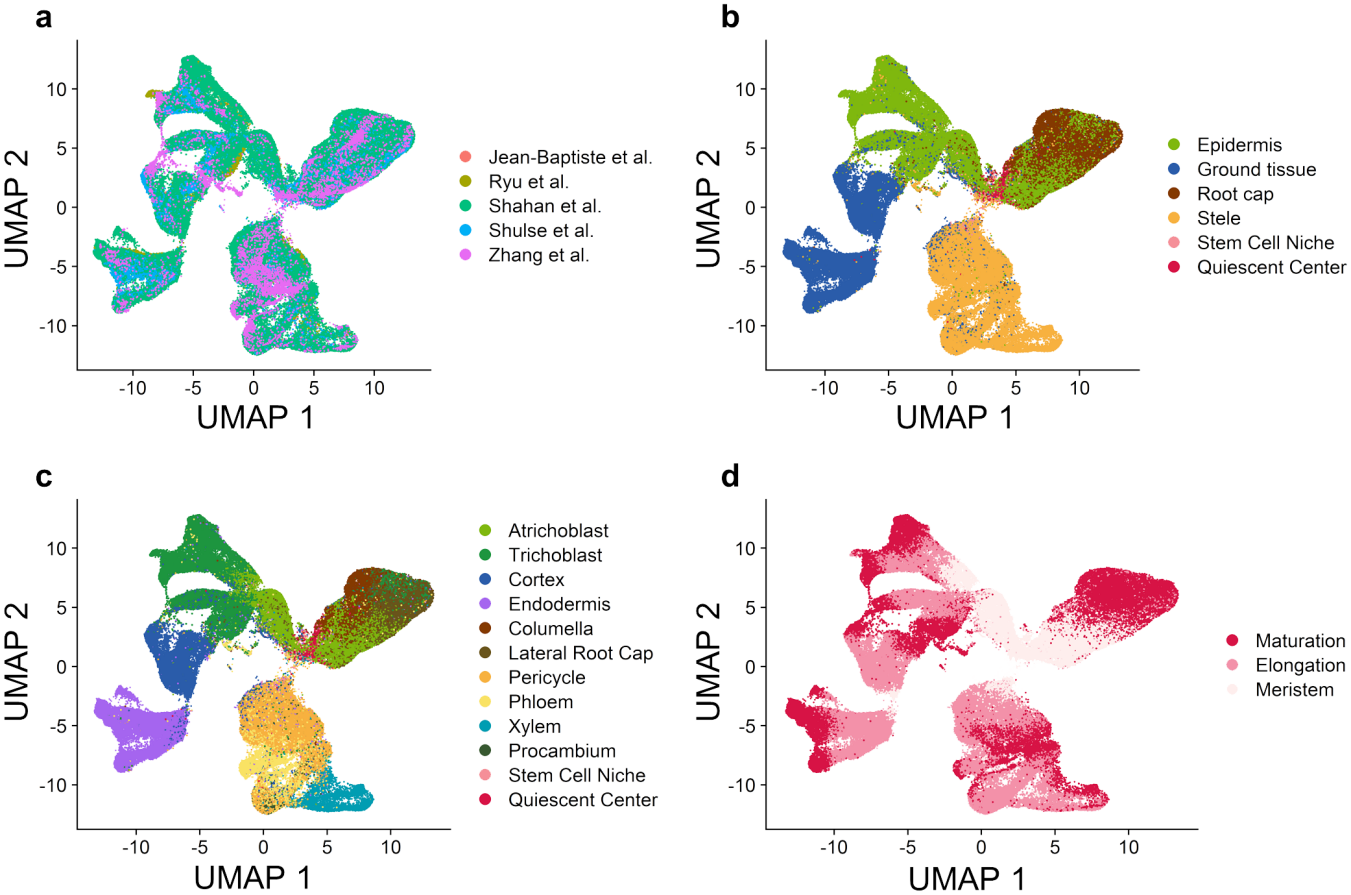
Integration of gene expression profiles from multiple studies reveals major cell types in wild-type Arabidopsis root tissue. **(A)** UMAP plot based on the integrated gene expression matrix from 28 scRNA-seq datasets. **(B)** UMAP plot with the annotation of four major branches and two stem cell types. **(C)** UMAP plot with the annotation of twelve cell types. **(D)** UMAP plot with the annotation of three root developmental stages.

Originally, a total of 150,697 cells were obtained from 28 published scRNA-seq datasets of wild-type Arabidopsis root tissue from five studies (**Supplementary Table S1**). These gene-cell expression matrices were combined with batch effect removed (see Materials and Methods) to construct a unified gene expression matrix containing 12,191 genes and 128,549 cells. The UMAP (Uniform Manifold Approximation and Projection) plot shows clearly the removal of batch effects of individual heterologous datasets (**Figure 2A**). Next, we designed three strategies (see Materials and Methods) to annotate these cells, which defined four root tissue branches and two major cell types (**Figure 2B**) and 12 cell types (**Figure 2C**). It could be seen that these cells continuously divide and differentiate to form four main branches, including the root cap tissue composed of lateral root cap and columella cells; the epidermis tissue composed of trichoblast (hair) and atrichoblast (non-hair) cells; the ground tissue composed of cortex and endodermis cells; and the stele tissue composed of phloem, xylem, procambium, and pericycle cells. Referred to published Arabidopsis root bulk RNA-seq data (Brady et al., 2007; Li et al., 2016), each cell could be further categorized into the three phases of root development: meristem, elongation, and maturation. Stem cells are mainly stored in the stem cell niche (SCN) at the root tip (Benfey, 2016), which contains the quiescent center (QC) as well as its surrounding stem cells (Aichinger et al., 2012). **Figure 2D** reflects the temporal developmental process. All cells start from stem cells, then gradually differentiate along the branches and undergo growth processes in the elongation zone, ultimately maturing at the branch tip. Overall, these results suggest that the gene expression atlas at the single-cell resolution highlights existing root development process and can help to explain the interconnections between and within different cell types.

### 3.3 An atlas of poly(A) sites at single-cell resolution in Arabidopsis roots

**Figure 3:**
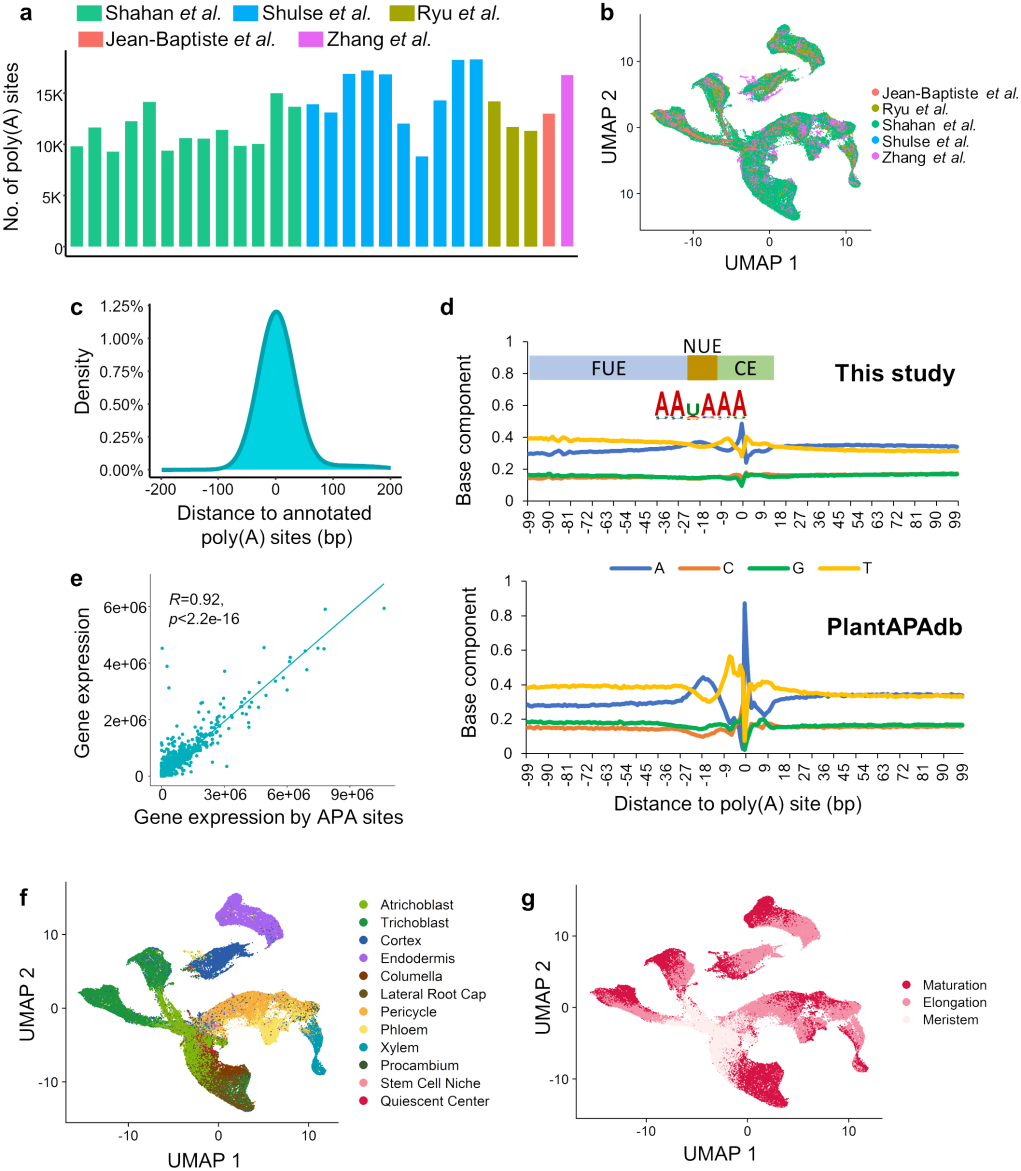
Atlas of poly(A) sites at single-cell resolution in Arabidopsis roots. **(A)** Number of 3′ UTR poly(A) sites identified from each of the 28 scRNA-seq datasets from five studies. **(B)** UMAP plot based on the integrated poly(A) site expression matrix. **(C)** Distribution of distance from identified single-cell poly(A) sites to annotated poly(A) sites in PlantAPAdb. **(D)** Nucleotide compositions of the sequences surrounding poly(A) sites. The top panel presents the single nucleotide profile of 3′ UTR poly(A) sites identified in this study. The bottom panel presents single nucleotide profile of 3′ UTR poly(A) sites collected from PlantAPAdb. X-axis denotes the position and 0 is the position of the poly(A) site, *i.e.*, the cleavage site (CS). The poly(A) signal regions around poly(A) sites in plants were marked, including the far upstream element (FUE) located at -100 ~ -25 bp, the near upstream element (NUE) located at -25 ~ -15 bp, and the cleavage element (CE) located at -15 ~ +15 bp. The sequence logo of hexamers in the NUE shows the most dominant poly(A) signal AAUAAA. **(E)** Scatterplot of correlation between gene expression from the gene-cell matrix and the gene expression by summarizing poly(A) sites in each gene. Each dot represents a single cell. **(F)** UMAP plot based on the poly(A) site matrix with the annotation of twelve cell types. **(G)** UMAP plot based on the poly(A) site matrix with the annotation of three developmental stages.

Single-cell poly(A) sites were identified and quantified from each of the 28 RNA-seq datasets, with the number of sites ranging from 8,825 to 18,298 (**Figure 3A**). Next, we proposed a integration strategy to integrate the poly(A) site profiles from these 28 batches, and a unified poly(A) site list of Arabidopsis root with the expression profile in each sample was obtained (see Materials and Methods). Finally, a poly(A) site expression matrix with 29,784 poly(A) sites in 128,134 cells was constructed, with batch effect removed (**Figure 3B**). To examine the accuracy of the obtained poly(A) sites, we compared the integrated poly(A) site list with annotated 3′ UTR poly(A) sites of Arabidopsis from PlantAPAdb (Zhu et al., 2020). Single-cell poly(A) sites identified in this study were located near annotated poly(A) sites in PlantAPAdb, with up to 77.5% poly(A) sites located within 100 bp of annotated sites (**Figure 3C**). The general distribution pattern of nucleotide composition surrounding poly(A) sites identified in this study was similar to that in PlantAPAdb (**Figure 3D**). Moreover, three typical plant poly(A) signal regions, including far upstream element (FUE), near upstream element (NUE), and cleavage element (CE) (Loke et al., 2005), can be revealed from the base compositions surrounding poly(A) sites (**Figure 3D**). Polyadenylation is guided by poly(A) signals in these regions that are recognized by core polyadenylation factors. The NUE located at -25 ~ -15 bp upstream of the poly(A) site contains the most conserved poly(A) signal AAUAAA (**Figure 3D**). The FUE is located at -100 ~ -25 bp upstream of the poly(A) site and is typically U-rich. The cleavage element is located at -15 ~ +15 bp around the poly(A) site, with YA (Y=C/U) at the cleavage site situated within a U-rich region. Further, we examined the poly(A) site quantification by comparing the expression profile from poly(A) sites with that from the gene-cell expression matrix. We summed the expression levels of all poly(A) sites on a gene to represent the expression level of the gene. The single-cell expression profile based on the gene expression data was highly correlated with that based on the poly(A) site data (Pearson’ correlation coefficient = 0.92, *P* value < 2.2e-16) (**Figure 3E**), demonstrating the high confidence of our poly(A) site data. Moreover, through the UMAP plot based on the expression matrix of poly(A) sites, it can be found that the distribution of cell clusters with the cell annotation results based on the APA profile is similar to that based on gene expression profile (**Figures 3F, G**). Cells from the same tissue branches are grouped together, and cells gradually matures along the branches.

### 3.4 Differential analysis of APA dynamics across cell types

**Figure 4:**
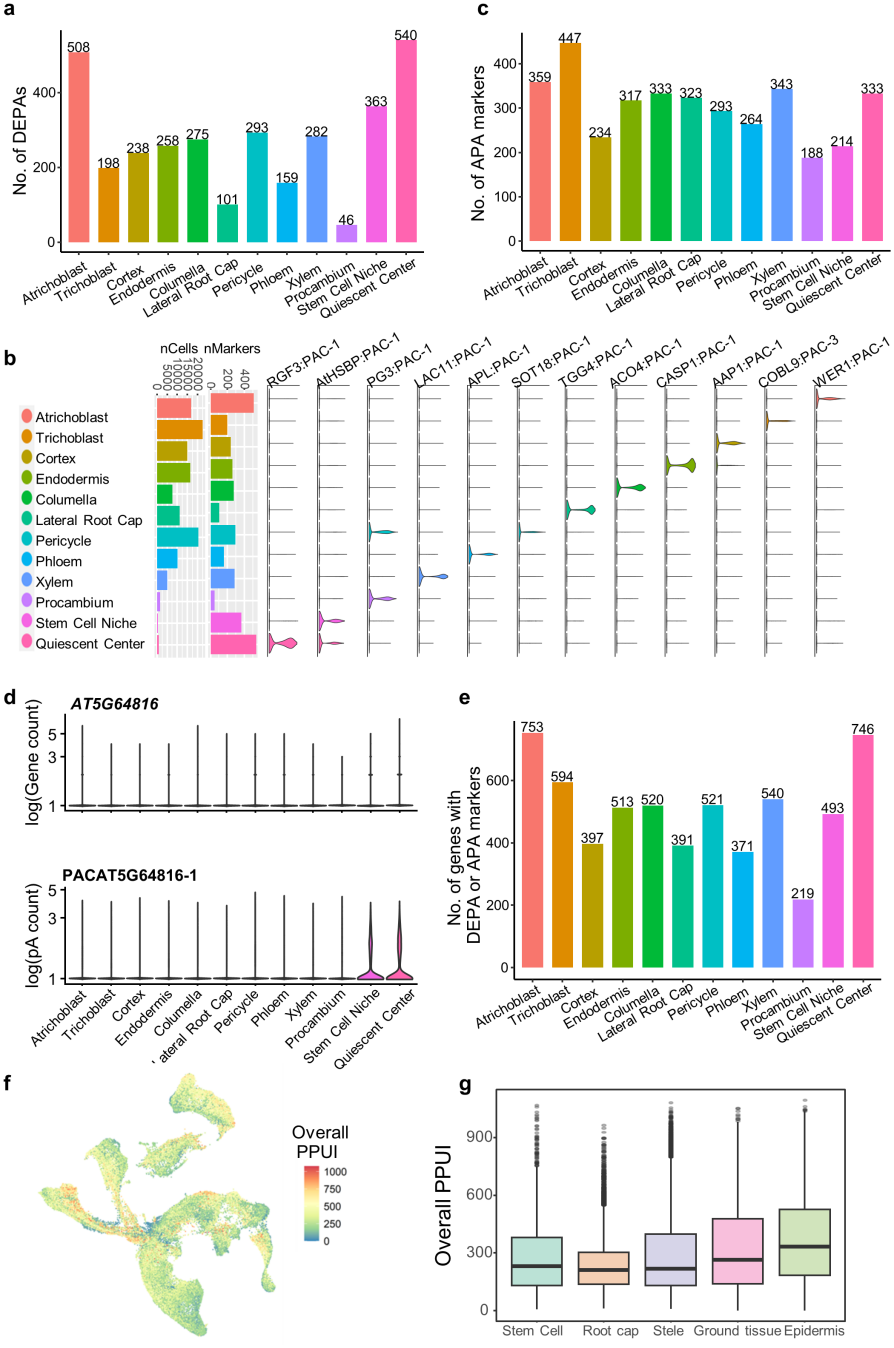
Differential analysis of APA dynamics across cell types in Arabidopsis roots. **(A)** Number of differentially expressed poly(A) sites (DEPAs) identified in each of the twelve cell types. **(B)** Representative DEPAs for the twelve cell types. Bar plots show the number of cells and number of identified DEPAs in each cell type. Violin plots show the expression profiles of representative DEPAs of different genes in different cell types. **(C)** Number of APA markers identified in each of the twelve cell types. **(D)** Violin plots showing the gene expression profile (top) and the DEPA expression profile (bottom) of the gene *AT5G64816* in different cell types. **(E)** Number of non-redundant genes with DEPAs or APA markers in each cell type. **(F)** UMAP plot showing the overall distribution of the proximal poly(A) site usage index (PPUI) of all the genes in a single cells. **(G)** Boxplot showing the overall distribution of PPUI of all the genes in different cell populations.

After integrating the panorama of poly(A) sites for Arabidopsis root cells, differential usage of poly(A) sites for four main tissue types and twelve cell types was assessed, and DEPAs were obtained for each cell type (see Materials and Methods). A total of 3,261 cell type-specific DEPAs were obtained for the twelve cell types, ranging from 46 to 540 DEPAs for each cell type (**Figure 4A**; **Supplementary Table S2**). Particularly, DEPAs of representative marker genes in root cell types were also discovered (**Figure 4B**), such as *WEREWOLF 1* (*WER1)* specifically expressed in atrichoblast (Lee and Schiefelbein, 1999); *COBRA-LIKE 9* (*COBL9*) specifically expressed in trichoblast (Kamiya et al., 2016); *ALTERED PHLOEM DEVELOPMENT* (*APL*) specifically expressed in phloem (Bonke et al., 2003); *CASPARIAN STRIP MEMBRANE DOMAIN PROTEIN 1* (*CASP1*) specifically expressed in endodermis (Roppolo et al., 2011). DEPAs located in these genes were highly enriched in relevant cell types. Moreover, it can be found that there is no significant relationship between the number of cells in a cell type and the number of DEPAs (**Figure 4B**). For example, atrichoblast and pericycle contain more cells and more DEPAs, whereas quiescent center contains very few cells yet with the highest number of DEPAs. This may suggest that many poly(A) sites are specifically expressed in nondividing quiescent cells of the root tip tissue during Arabidopsis root cell development, regulating the differentiation of the tissue.

We further investigated the dynamic changes in the usage of APA sites across different cell types and developmental periods. To this end, we constructed an APA ratio matrix (see Materials and Methods), and obtained 3648 APA markers with differential APA usages across 12 cell types (**Supplementary Table S3**; **Figure 4C**). Specifically, some APA markers are expressed in a specific cell type, but their corresponding genes do not exhibit specific gene expression in that cell type. For example, the proximal poly(A) site of *AT5G64816* is up-regulated in stem cells (stem cell niche and quiescent center), but the gene is expressed at an average level across all cell types (**Figure 4D**). These APA markers are particularly useful for discovering and annotating cell types that are difficult to identify based solely on gene level analysis, which are important in analyzing the gene regulatory role of APA in cell differentiation and tissue development in Arabidopsis. Particularly, DEPA genes and APA markers show a relatively small overlap (**Figure 4E**). For example, 508 DEPAs and 359 APA markers were found specific to Atrichoblast, and up to 753 genes were retained after removing redundancy (**Figure 4E**). Finally, 219 to 753 cell type-specific genes with DEPAs or APA markers were obtained for different cell types (**Figure 4E**), which represent an important gene resource related to APA dynamics in Arabidopsis single cells.

To further investigate the dynamic pattern of APA usages during Arabidopsis development, we summarized PPUI score of all genes in each cell to represent the usage of proximal poly(A) for each cell. PPUI scores are evenly distributed in most of the cells, but appear to be aggregated in some tissues (**Figure 4F**). PPUI scores in individual tissues were further compared (**Figure 4G**). It can be seen that PPUI scores were significantly decreased on root cap tissues compared with stem cells (stem cell niche and quiescent center), while PPUI scores in other tissues were similar to that of stem cells. This suggests that during the development process of Arabidopsis root, stem cells are more likely to be more inclined to use the proximal poly (A) site. As the cells gradually mature, the majority of mature cells that differentiate into root cap gradually decrease their use of the proximal poly(A) site, indicating an increasing trend of 3′ UTR lengthening.

### 3.5 APA dynamics between trichoblast and atrichoblast

**Figure 5:**
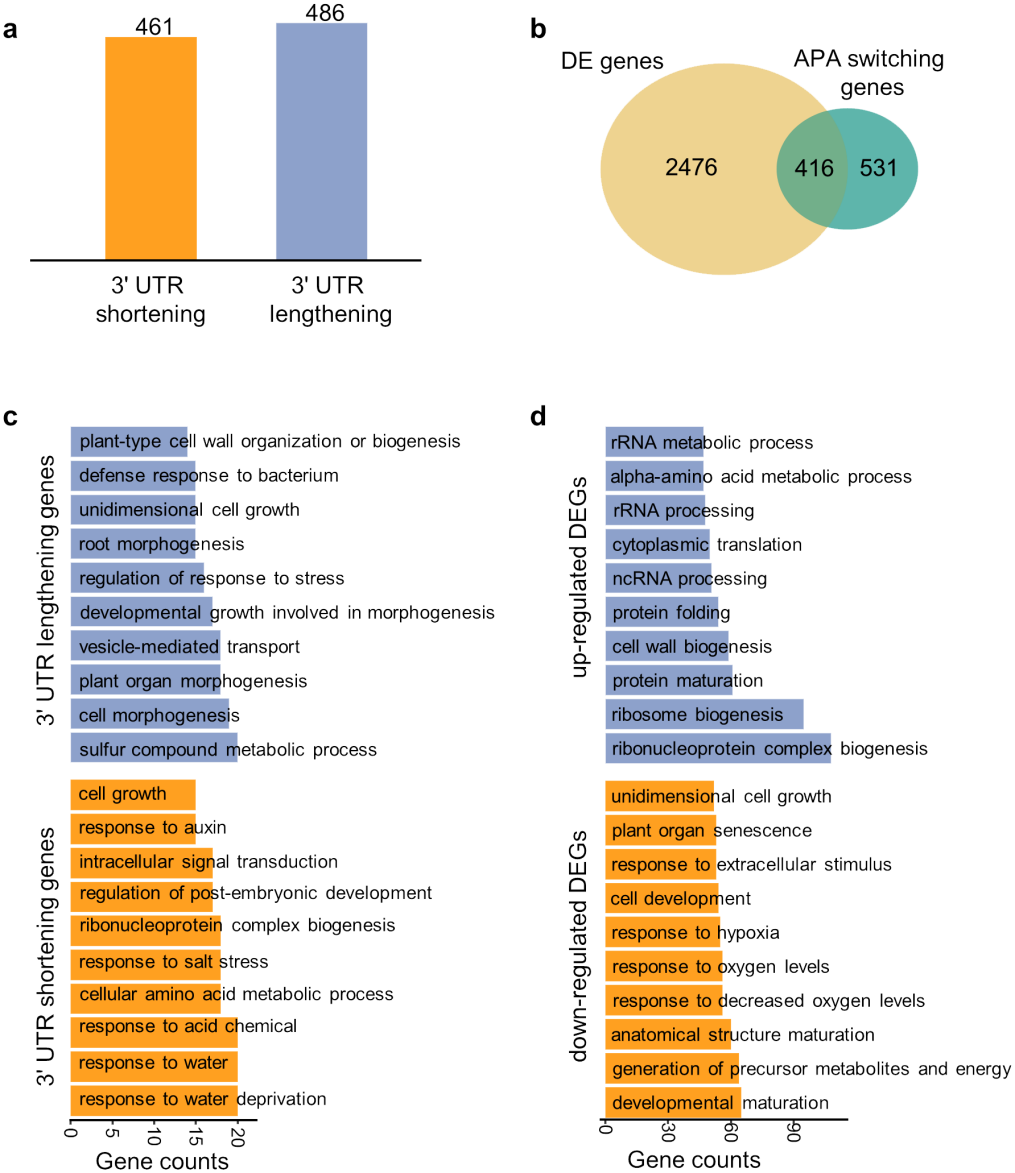
Differential analysis of APA dynamics between trichoblast and atrichoblast. **(A)** Number of APA switching genes with 3′ UTR lengthening or shortening in atrichoblast. **(B)** Venn diagram showing the overlap between differentially expressed genes (DEGs) and APA switching genes. **(C)** Gene ontology analysis of the APA switching genes with 3′ UTR lengthening or shortening in atrichoblast. **(D)** Gene ontology analysis of the DEGs up-regulated or down-regulated in atrichoblast.

Next, we focused on the epidermis tissue and explored the difference in APA dynamics between trichoblast (hair) and atrichoblast (non-hair). Firstly, APA markers that are differentially used between trichoblast and atrichoblast were identified to obtain genes with APA switching between cell types. We adopted the relative 3′ UTR length weighted by number of read counts to further determine the direction of switching (see Materials and Methods). A total of 486 genes with 3′ UTR lengthening in atrichoblast and 461 genes with 3′ UTR shortening in atrichoblast were obtained, respectively (**Figure 5A**). In contrast, 2,892 differentially expressed genes (DEGs) between trichoblast and atrichoblast were obtained based on the gene expression profile, with 1,335 up-regulated genes and 1,557 down-regulated genes in atrichoblast. There is moderate overlap between DEGs and APA switching genes, with 44% (416/947) of APA genes overlapping with DEGs (**Figure 5B**). GO (Gene Ontology) analysis was performed on these genes to investigate their biological functions (**Supplementary Tables S4**, **S5**). Genes with APA switching were enriched in biological terms specific to the two cell types (**Figure 5C**). The 3′ UTR lengthened genes in atrichoblast are involved in cell growth and cell morphogenesis which are mainly associated with morphological changes in cells, demonstrating the biological process of plant epidermal cell differentiation. In contrast, the 3′ UTR shortened APA switching genes in atrichoblast are involved in the response to water deprivation and response to acid chemical which are mainly related to stress responses under various conditions, demonstrating the adaptive differentiation of plant fibers to the environment. We also conducted GO analysis on DEGs and found that the GO term were different from the APA results (**Figure 5D**). Specifically, up-regulated DEGs were enriched in several common GO terms such as rRNA metabolic process, protein folding, and protein maturation. Down-regulated DEGs were enriched in biological processes related to stress responses such as hypoxia. This result indicates that APA switching genes and differentially expressed genes are relatively independent gene sets, representing different layers of gene expression regulation.

### 3.6 Inclusion of APA markers for annotating cell types

**Figure 6:**
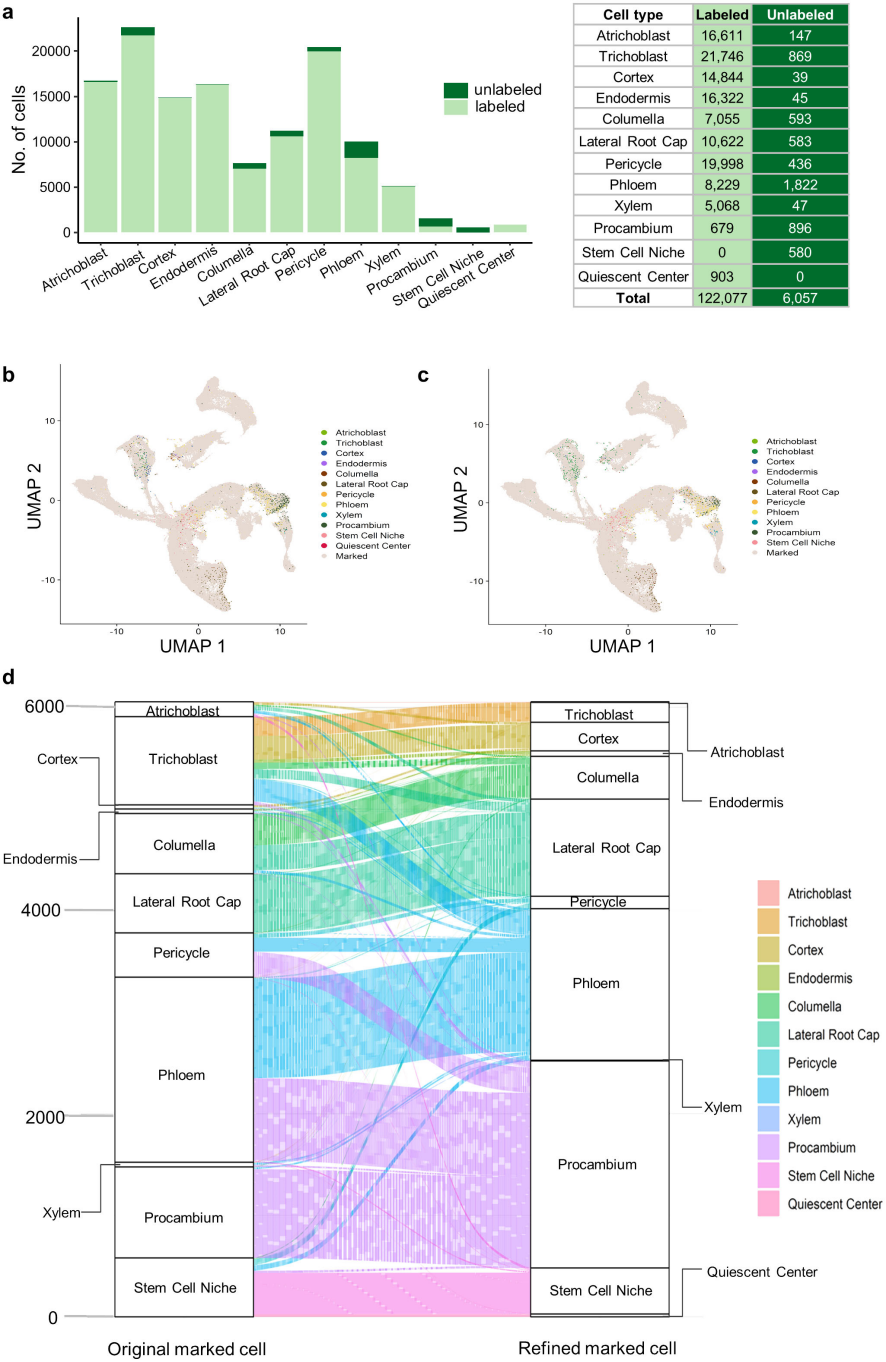
Inclusion of APA markers for annotating cell types. **(A)** Number of labeled and unlabeled cells in each cell type. A total of 128,134 cells present in both the integrated gene expression matrix and APA matrix were counted. Initial cell labels of unlabeled cells were determined by calculating Pearson’s correlation coefficient between the gene expression profile of each unlabeled cell and the reference profile of the labeled cell types. **(B)** UMAP plot showing unlabeled cells with the cell annotation after correction with DEPAs and APA markers. **(C)** UMAP plot showing unlabeled cells with the cell annotation based on the gene-cell expression matrix alone. **(D)** Alluvial plot showing the re-assignment of cell labels for unlabeled cells after correction with DEPAs and APA markers.

We integrated three strategies to assign cell type label for each root cell based on the single-cell gene expression profile alone (see Materials and Methods), but 6,057 cells (from 128,134 cells) were still remained unannotated (called unlabeled cells) (**Figure 6A**). Our above analysis indicates that APA information can represent the differential use of different APA isoforms of the same gene (**Figures 4D**, **5B**), which may help to supplement gene expression information to improve cell identity determination. Therefore, we integrated the annotation results based on gene expression, DEPAs and APA markers (see Materials and Methods) to obtain new identities for these unlabeled cells (**Figure 6B**). For comparison, we roughly annotated these unlabeled cells by calculating Pearson’s correlation coefficient between the gene expression profile of each unlabeled cell and the reference profile of the labeled cell types (**Figure 6C**). We then used the alluvial map to further demonstrate the refining of cell identities after the inclusion of the APA profile (**Figure 6D**), and found some interesting observations. For example, 147 cells originally classified as atrichoblast based solely on the gene expression level were reassigned to other cell types such as trichoblast or cortex after correction with APA information. Such reassignment not only indicates that some single cells may have multiple developmental potentials, but also reflects that the boundaries between some cell types may be less clear than expected. Specifically, there are the greatest number of genes with differential APA usage in atrichoblast (**Figure 4E**), suggesting that APA dynamics may be the most intense in this cell type. As such, APA information could be particularly important for determining the identity of these atrichoblast-like cells. Overall, the integration of the complementary information of gene expression and APA profiles can help annotate cell types and reveal subtle differences between cell types, which is of significant importance for the study of cell fate determination and tissue development.

## 4 Discussion

ScRNA-seq technologies have been widely used to reveal the diversity and complexity of cells, especially for gene expression research at single-cell resolution. ScRNA-seq has shown great potential in the field of biomedical applications (Chen et al., 2021; Song et al., 2022; Feng et al., 2024), however, its application in plants far lags behind due to technical limitations in isolating plant cells. It was not until 2019 that the feasibility and effectiveness of scRNA-seq in plants were confirmed (Denyer et al., 2019; Jean-Baptiste et al., 2019; Ryu et al., 2019; Shulse et al., 2019; Zhang et al., 2019), and thereafter, a large number of studies on scRNA-seq in plants began to emerge (Luo et al., 2020; McFaline-Figueroa et al., 2020; Wendrich et al., 2020; Bawa et al., 2022). Although these studies have utilized scRNA-seq to analyze plant gene expression regulation at the single-cell level, they were limited to the gene level, and there is still a lack of research with single-cell resolution on the transcript-isoform level. As an essential post-transcriptional mechanism for regulating gene expression, APA plays important roles in regulating flowering time, seed dormancy, root development, and stress response in plants (Cyrek et al., 2016; Lin et al., 2017; Ma et al., 2022; Yu et al., 2022). ScRNA-seq data not only provide the gene expression profile, but also the usages of transcript isoforms in single cells, providing richer information for characterizing single-cell transcriptomes. In this study, we comprehensively compiled a single-cell APA atlas of Arabidopsis roots covering 12 cell types in four major tissue branches and three developmental stages. Moreover, we quantified the dynamic APA usages in single cells and identified APA markers across tissues and cell types. By integrating both the APA information and the gene expression profile, we improved cell type annotations of single root cells.

There are currently many computational methods for annotating cell types based on gene expression (Huang et al., 2021; Qi et al., 2020; Zhao et al., 2020; Lin et al., 2023; Xu et al., 2023, 2024), but none of them is applicable to all situations. In this study, we improved the reliability of cell type annotation by combining three strategies, using annotations with the same cell label in at least two strategies as the final annotation for each cell. This approach combines the unsupervised method and methods based on reference gene expression, reducing the possibility of cells being incorrectly annotated by a single method. But admittedly, this approach will lead to an increase in the number of unlabeled cells. However, in this study, we focused on the transcriptome differences between cell types, so we chose to use more accurate cell annotation results to avoid bias caused by the introduction of incorrectly labeled cells. Moreover, it is worth noting that these unlabeled cells are also likely to be indistinguishable solely based on the gene expression profile. Usages of APA sites of a gene represent the expression of different transcript isoforms of the same gene, which may provide complementary information of gene expression for determining cell identity (**Figure 6B**). We found that after using APA information for cell type annotation, many unlabeled cells were labeled, while many cells’ cell type labels obtained based on gene expression and APA were different (**Figure 6D**). This may be because our annotation considers more similar sub-cell types rather than main cell types, and the differentiation between sub-cell types may be subtle, making cell labels susceptible to changes due to different input information. In the future, more information could be included, such as experimentally-verified marker genes or information from additional omics (e.g., spatial transcriptomics), to validate cell type annotation results or better define cell identity.

At present, there are many methods for integrating gene expression data from different studies or batches (Butler et al., 2018; Tran et al., 2020; Yang et al., 2021; Xiong et al., 2022). However, these methods are not suitable for integrating APA sites from different studies. This is because APA sites are represented by genomic interval information, and the intervals of the same APA site may not be completely same across different data sources. We designed a method to combine APA sites from different scRNA-seq batches. This method iteratively merged APA site intervals from different batches to the same site, ensuring the accuracy of APA site annotation. This approach can unify the annotation of the same APA sites from different batches, while also distinguishing adjacent or even overlapping but different APA sites. The nucleic acid distribution and poly(A) signals surrounding the combined APA sites confirm the effectiveness of our APA site identification and integration pipeline (**Figure 3D**).

The collection of APA data from different studies provides a high-quality data foundation for analyzing APA dynamics in single cells. We identified three kinds of APA markers, including DEPAs based on APA site expression, APA markers based on the APA ratios, and APA switching genes based on 3′ UTR length. DEPA identification is similar to traditional DEG analysis, while considering the expression level of a single APA site. By integrating the expression levels of all poly(A) sites in a gene, the gene expression level can be approximated, further verifying the accuracy of poly(A) site quantification (**Figure 3E**). The PPUI indicator represents the usage of the proximal poly(A) site by considering expression levels of different APA sites of a gene, allowing for the comparison of proximal poly(A) usage of the same gene among different cell types at the gene level (**Figure 4F**). Although the PPUI indicator, to some extent, reflects the changes in 3′ UTR length (the higher the PPUI, the shorter the 3′ UTR), it ignores the 3′ UTR length and the expression level of individual sites within a gene. Therefore, we further calculated the weighted average 3′ UTR length for each gene to more accurately characterize the significance of the 3′ UTR length changes of genes in different cell types (**Figure 5A**). These diverse APA dynamic characterization methods provide a more comprehensive APA dynamic profile across different cell types in Arabidopsis roots.

## Data Availability

The original contributions presented in the study are included in the article/**Supplementary Material**, further inquiries can be directed to the corresponding authors. All the scripts used for analysis in this study are available at https://github.com/BMILAB/root_scAPA_atlas.

## References

[B1] Aibar S. González-Blas C. B. Moerman T. Huynh-Thu V. A. Imrichova H. Hulselmans G.. (2017). SCENIC: single-cell regulatory network inference and clustering. Nat. Methods 14, 1083. doi: 10.1038/nmeth.4463 28991892 PMC5937676

[B2] Aichinger E. Kornet N. Friedrich T. Laux T. (2012). Plant stem cell niches. Annu. Rev. Plant Biol. 63, 615–636. doi: 10.1146/annurev-arplant-042811-105555 22404469

[B3] Arzalluz-Luque A. Conesa A. (2018). Single-cell RNAseq for the study of isoforms-how is that possible? Genome Biol. 19, 110. doi: 10.1186/s13059-018-1496-z 30097058 PMC6085759

[B4] Bawa G. Liu Z. Yu X. Qin A. Sun X. (2022). Single-cell RNA sequencing for plant research: insights and possible benefits. Int. J. Mol. Sci. 23. doi: 10.3390/ijms23094497 PMC9100049 35562888

[B5] Benfey P. N. (2016). “Chapter Three - Defining the Path from Stem Cells to Differentiated Tissue,” in Current Topics in Developmental Biology. Ed. Wassarman P. M. (New York: Academic Press), 35–43. 10.1016/bs.ctdb.2015.12.002 26970612

[B6] Birnbaum K. Jung J. W. Wang J. Y. Lambert G. M. Hirst J. A. Galbraith D. W.. (2005). Cell type–specific expression profiling in plants via cell sorting of protoplasts from fluorescent reporter lines. Nat. Methods 2, 615–619. doi: 10.1038/nmeth0805-615 16170893

[B7] Birnbaum K. D. Kussell E. (2011). Measuring cell identity in noisy biological systems. Nucleic Acids Res. 39, 9093–9107. doi: 10.1093/nar/gkr591 21803789 PMC3241637

[B8] Bonke M. Thitamadee S. Mähönen A. P. Hauser M.-T. Helariutta Y. (2003). APL regulates vascular tissue identity in Arabidopsis. Nature 426, 181–186. doi: 10.1038/nature02100 14614507

[B9] Brady S. M. Orlando D. A. Lee J.-Y. Wang J. Y. Koch J. Dinneny J. R.. (2007). A high-resolution root spatiotemporal map reveals dominant expression patterns. Science 318, 801–806. doi: 10.1126/science.1146265 17975066

[B10] Bruex A. Kainkaryam R. M. Wieckowski Y. Kang Y. H. Bernhardt C. Xia Y.. (2012). A gene regulatory network for root epidermis cell differentiation in arabidopsis. PloS Genet. 8. doi: 10.1371/journal.pgen.1002446 PMC3257299 22253603

[B11] Butler A. Hoffman P. Smibert P. Papalexi E. Satija R. (2018). Integrating single-cell transcriptomic data across different conditions, technologies, and species. Nat. Biotechnol. 36, 411–420. doi: 10.1038/nbt.4096 29608179 PMC6700744

[B12] Chen S. Zhu G. Yang Y. Wang F. Xiao Y.-T. Zhang N.. (2021). Single-cell analysis reveals transcriptomic remodellings in distinct cell types that contribute to human prostate cancer progression. Nat. Cell Biol. 23, 87–98. doi: 10.1038/s41556-020-00613-6 33420488

[B13] Cyrek M. Fedak H. Ciesielski A. Guo Y. Sliwa A. Brzezniak L.. (2016). Seed dormancy in arabidopsis is controlled by alternative polyadenylation of DOG1. Plant Physiol. 170, 947–955. doi: 10.1104/pp.15.01483 26620523 PMC4734566

[B14] Denyer T. Ma X. Klesen S. Scacchi E. Nieselt K. Timmermans M. C. P. (2019). Spatiotemporal developmental trajectories in the arabidopsis root revealed using high-throughput single-cell RNA sequencing. Dev. Cell 48, 840–852.e845. doi: 10.1016/j.devcel.2019.02.022 30913408

[B15] Dobin A. Davis C. A. Schlesinger F. Drenkow J. Zaleski C. Jha S.. (2013). STAR: ultrafast universal RNA-seq aligner. Bioinformatics 29, 15–21. doi: 10.1093/bioinformatics/bts635 23104886 PMC3530905

[B16] Efroni I. Ip P.-L. Nawy T. Mello A. Birnbaum K. D. (2015). Quantification of cell identity from single-cell gene expression profiles. Genome Biol. 16. doi: 10.1186/s13059-015-0580-x PMC4354993 25608970

[B17] Feng D.-C. Zhu W.-Z. Wang J. Li D.-X. Shi X. Xiong Q.. (2024). The implications of single-cell RNA-seq analysis in prostate cancer: unraveling tumor heterogeneity, therapeutic implications and pathways towards personalized therapy. Military Med. Res. 11, 21. doi: 10.1186/s40779-024-00526-7 PMC11007901 38605399

[B18] Fu H. Yang D. Su W. Ma L. Shen Y. Ji G.. (2016). Genome-wide dynamics of alternative polyadenylation in rice. Genome Res. 26, 1753–1760. doi: 10.1101/gr.210757.116 27733415 PMC5131826

[B19] Gao Y. Li L. Amos C. I. Li W. (2021). Analysis of alternative polyadenylation from single-cell RNA-seq using scDaPars reveals cell subpopulations invisible to gene expression. Genome Research 31 (10), 1856–1866. doi: 10.1101/gr.271346.120 34035046 PMC8494218

[B20] Gruber A. J. Zavolan M. (2019). Alternative cleavage and polyadenylation in health and disease. Nat. Rev. Genet. 20, 1. doi: 10.1038/s41576-019-0145-z 31267064

[B21] Huang Q. Liu Y. Du Y. Garmire L. X. (2021). Evaluation of cell type annotation R packages on single-cell RNA-seq data. Genomics Proteomics Bioinf. 19 (2), 267–281. doi: 10.1016/j.gpb.2020.07.004 PMC8602772 33359678

[B22] Jean-Baptiste K. McFaline-Figueroa J. L. Alexandre C. M. Dorrity M. W. Saunders L. Bubb K. L.. (2019). Dynamics of gene expression in single root cells of arabidopsis thaliana. Plant Cell 31, 993–1011. doi: 10.1105/tpc.18.00785 30923229 PMC8516002

[B23] Kamiya M. Higashio S.-Y. Isomoto A. Kim J.-M. Seki M. Miyashima S.. (2016). Control of root cap maturation and cell detachment by BEARSKIN transcription factors in Arabidopsis. Development 143, 4063–4072. doi: 10.1242/dev.142331 27803060

[B24] Kim N. Chung W. Eum H. H. Lee H. O. Park W. Y. (2019). Alternative polyadenylation of single cells delineates cell types and serves as a prognostic marker in early stage breast cancer. PloS One 14, e0217196. doi: 10.1371/journal.pone.0217196 31100099 PMC6524824

[B25] Kolodziejczyk A. A. Kim J. K. Svensson V. Marioni J. C. Teichmann S. A. (2015). The technology and biology of single-cell RNA sequencing. Mol. Cell 58, 610–620. doi: 10.1016/j.molcel.2015.04.005 26000846

[B26] Korsunsky I. Millard N. Fan J. Slowikowski K. Zhang F. Wei K.. (2019). Fast, sensitive and accurate integration of single-cell data with Harmony. Nat. Methods 16, 1289–1296. doi: 10.1038/s41592-019-0619-0 31740819 PMC6884693

[B27] Lee M. M. Schiefelbein J. (1999). WEREWOLF, a MYB-related protein in Arabidopsis, is a position-dependent regulator of epidermal cell patterning. Cell 99, 473–483. doi: 10.1016/S0092-8674(00)81536-6 10589676

[B28] Li S. Yamada M. Han X. Ohler U. Benfey P. N. (2016). High-resolution expression map of the arabidopsis root reveals alternative splicing and lincRNA regulation. Dev. Cell 39, 508–522. doi: 10.1016/j.devcel.2016.10.012 27840108 PMC5125536

[B29] Lin J.-L. Chen L. Wu W.-K. Guo X.-X. Yu C.-H. Xu M.. (2023). Single-cell RNA sequencing reveals a hierarchical transcriptional regulatory network of terpenoid biosynthesis in cotton secretory glandular cells. Mol. Plant 16, 1990–2003. doi: 10.1016/j.molp.2023.10.008 37849250

[B30] Lin J. Xu R. Wu X. Shen Y. Li Q. Q. (2017). Role of cleavage and polyadenylation specificity factor 100: anchoring poly(A) sites and modulating transcription termination. Plant J. 91, 829–839. doi: 10.1111/tpj.13611 28621907

[B31] Loke J. C. Stahlberg E. A. Strenski D. G. Haas B. J. Wood P. C. Li Q. Q. (2005). Compilation of mRNA polyadenylation signals in arabidopsis revealed a new signal element and potential secondary structures. Plant Physiol. 138, 1457–1468. doi: 10.1104/pp.105.060541 15965016 PMC1176417

[B32] Luo C. Fernie A. R. Yan J. (2020). Single-cell genomics and epigenomics: technologies and applications in plants. Trends Plant Sci. 25, 1030–1040. doi: 10.1016/j.tplants.2020.04.016 32532595

[B33] Ma H. Cai L. Lin J. Zhou K. Li Q. Q. (2022). Divergence in the Regulation of the Salt Tolerant Response Between Arabidopsis thaliana and Its Halophytic Relative Eutrema salsugineum by mRNA Alternative Polyadenylation. Front. Plant Sci. 13. doi: 10.3389/fpls.2022.866054 PMC8993227 35401636

[B34] Marinov G. K. Williams B. A. McCue K. Schroth G. P. Gertz J. Myers R. M.. (2014). From single-cell to cell-pool transcriptomes: Stochasticity in gene expression and RNA splicing. Genome Res. 24, 496–510. doi: 10.1101/gr.161034.113 24299736 PMC3941114

[B35] McFaline-Figueroa J. L. Trapnell C. Cuperus J. T. (2020). The promise of single-cell genomics in plants. Curr. Opin. Plant Biol. 54, 114–121. doi: 10.1016/J.PBI.2020.04.002 32388018 PMC7971421

[B36] Patrick R. Humphreys D. T. Janbandhu V. Oshlack A. Ho J. W. K. Harvey R. P.. (2020). Sierra: discovery of differential transcript usage from polyA-captured single-cell RNA-seq data. Genome Biol. 21, 167. doi: 10.1186/s13059-020-02071-7 32641141 PMC7341584

[B37] Qi R. Ma A. Ma Q. Zou Q. (2020). Clustering and classification methods for single-cell RNA-sequencing data. Briefings Bioinf. 21, 1196–1208. doi: 10.1093/BIB/BBZ062 PMC7444317 31271412

[B38] Roppolo D. De Rybel B. Tendon V. D. Pfister A. Alassimone J. Vermeer J. E. M.. (2011). A novel protein family mediates Casparian strip formation in the endodermis. Nature 473, 380–383. doi: 10.1038/nature10070 21593871

[B39] Ryu K. H. Huang L. Kang H. M. Schiefelbein J. (2019). Single-cell RNA sequencing resolves molecular relationships among individual plant cells. Plant Physiol. 179, 1444–1456. doi: 10.1104/pp.18.01482 30718350 PMC6446759

[B40] Saliba A.-E. Westermann A. J. Gorski S. A. Vogel J. (2014). Single-cell RNA-seq: advances and future challenges. Nucleic Acids Res. 42, 8845–8860. doi: 10.1093/nar/gku555 25053837 PMC4132710

[B41] Shahan R. Hsu C.-W. Nolan T. M. Cole B. J. Taylor I. W. Greenstreet L.. (2022). A single-cell Arabidopsis root atlas reveals developmental trajectories in wild-type and cell identity mutants. Dev. Cell 57, 543–560.e549. doi: 10.1016/j.devcel.2022.01.008 35134336 PMC9014886

[B42] Shalek A. K. Satija R. Adiconis X. Gertner R. S. Gaublomme J. T. Raychowdhury R.. (2013). Single-cell transcriptomics reveals bimodality in expression and splicing in immune cells. Nature 498, 236–240. doi: 10.1038/nature12172 23685454 PMC3683364

[B43] Shulman E. D. Elkon R. (2019). Cell-type-specific analysis of alternative polyadenylation using single-cell transcriptomics data. Nucleic Acids Res. 47, 10027–10039. doi: 10.1093/nar/gkz781 31501864 PMC6821429

[B44] Shulse C. N. Cole B. J. Ciobanu D. Lin J. Yoshinaga Y. Gouran M.. (2019). High-throughput single-cell transcriptome profiling of plant cell types. Cell Rep. 27, 2241–2247.e2244. doi: 10.1016/j.celrep.2019.04.054 31091459 PMC6758921

[B45] Smith T. Heger A. Sudbery I. (2017). UMI-tools: modeling sequencing errors in Unique Molecular Identifiers to improve quantification accuracy. Genome Res. 27, 491–499. doi: 10.1101/gr.209601.116 28100584 PMC5340976

[B46] Song Y. Botvinnik O. B. Lovci M. T. Kakaradov B. Liu P. Xu J. L.. (2017). Single-cell alternative splicing analysis with expedition reveals splicing dynamics during neuron differentiation. Mol. Cell 67, 148–161.e145. doi: 10.1016/j.molcel.2017.06.003 28673540 PMC5540791

[B47] Song H. Weinstein H. N. W. Allegakoen P. Wadsworth M. H. Xie J. Yang H.. (2022). Single-cell analysis of human primary prostate cancer reveals the heterogeneity of tumor-associated epithelial cell states. Nat. Commun. 13, 141. doi: 10.1038/s41467-021-27322-4 35013146 PMC8748675

[B48] Stuart T. Butler A. Hoffman P. Hafemeister C. Papalexi E. Mauck W. M.. (2019). Comprehensive integration of single-cell data. Cell 177, 1888–1902.e1821. doi: 10.1016/j.cell.2019.05.031 31178118 PMC6687398

[B49] Thomas P. E. Wu X. Liu M. Gaffney B. Ji G. Li Q. Q.. (2012). Genome-wide control of polyadenylation site choice by CPSF30 in arabidopsis. Plant Cell 24, 4376–4388. doi: 10.1105/tpc.112.096107 23136375 PMC3531840

[B50] Tian B. Manley J. L. (2017). Alternative polyadenylation of mRNA precursors. Nat. Rev. Mol. Cell Biol. 18, 18–30. doi: 10.1038/nrm.2016.116 27677860 PMC5483950

[B51] Tran H. T. N. Ang K. S. Chevrier M. Zhang X. Lee N. Y. S. Goh M.. (2020). A benchmark of batch-effect correction methods for single-cell RNA sequencing data. Genome Biol. 21, 12. doi: 10.1186/s13059-019-1850-9 31948481 PMC6964114

[B52] Velten L. Anders S. Pekowska A. Jarvelin A. I. Huber W. Pelechano V.. (2015). Single-cell polyadenylation site mapping reveals 3’ isoform choice variability. Mol. Syst. Biol. 11, 812–812. doi: 10.15252/msb.20156198 26040288 PMC4501847

[B53] Wang Y. Navin N. E. (2015). Advances and applications of single-cell sequencing technologies. Mol. Cell 58, 598–609. doi: 10.1016/j.molcel.2015.05.005 26000845 PMC4441954

[B54] Wang T. Ye W. Zhang J. Li H. Zeng W. Zhu S.. (2023). Alternative 3′-untranslated regions regulate high-salt tolerance of Spartina alterniflora. Plant Physiol. 191, 2570–2587. doi: 10.1093/plphys/kiad030 36682816 PMC10069910

[B55] Wendrich J. R. Yang B. Vandamme N. Verstaen K. Smet W. Van de Velde C.. (2020). Vascular transcription factors guide plant epidermal responses to limiting phosphate conditions. Science 370. doi: 10.1126/science.aay4970 PMC7116379 32943451

[B56] Wolf F. A. Angerer P. Theis F. J. (2018). SCANPY: large-scale single-cell gene expression data analysis. Genome Biol. 19. doi: 10.1186/s13059-017-1382-0 PMC5802054 29409532

[B57] Wu X. Liu M. Downie B. Liang C. Ji G. Li Q. Q.. (2011). Genome-wide landscape of polyadenylation in Arabidopsis provides evidence for extensive alternative polyadenylation. Proc. Natl. Acad. Sciences U.S.A. 108, 12533–12538. doi: 10.1073/pnas.1019732108 PMC3145732 21746925

[B58] Wu X. Liu T. Ye C. Ye W. Ji G. (2021). scAPAtrap: identification and quantification of alternative polyadenylation sites from single-cell RNA-seq data. Briefings Bioinf. 22. doi: 10.1093/bib/bbaa273 33142319

[B59] Xing D. Zhao H. Xu R. Li Q. Q. (2008). Arabidopsis PCFS4, a homologue of yeast polyadenylation factor Pcf11p, regulates FCA alternative processing and promotes flowering time. Plant J. 54, 899–910. doi: 10.1111/j.1365-313X.2008.03455.x 18298670

[B60] Xiong L. Tian K. Li Y. Ning W. Gao X. Zhang Q. C. (2022). Online single-cell data integration through projecting heterogeneous datasets into a common cell-embedding space. Nat. Commun. 13, 6118. doi: 10.1038/s41467-022-33758-z 36253379 PMC9574176

[B61] Xu J. Huang D.-S. Zhang X. (2024). scmFormer integrates large-scale single-cell proteomics and transcriptomics data by multi-task transformer. Advanced Sci. 11, 2307835. doi: 10.1002/advs.202307835 PMC11109621 38483032

[B62] Xu J. Zhang A. Liu F. Chen L. Zhang X. (2023). CIForm as a Transformer-based model for cell-type annotation of large-scale single-cell RNA-seq data. Briefings Bioinf. 24. doi: 10.1093/bib/bbad195 37200157

[B63] Yang Y. Li G. Qian H. Wilhelmsen K. C. Shen Y. Li Y. (2021). SMNN: batch effect correction for single-cell RNA-seq data via supervised mutual nearest neighbor detection. Briefings Bioinf. 22, bbaa097. doi: 10.1093/bib/bbaa097 PMC8324985 32591778

[B64] Ye W. Lian Q. Ye C. Wu X. (2023). A survey on methods for predicting polyadenylation sites from DNA sequences, bulk RNA-seq, and single-cell RNA-seq. Genomics Proteomics Bioinf. 21, 63–79. doi: 10.1016/j.gpb.2022.09.005 PMC10372920 36167284

[B65] Ye W. Liu T. Fu H. Ye C. Ji G. Wu X.. (2021). movAPA: modeling and visualization of dynamics of alternative polyadenylation across biological samples. Bioinformatics 37, 2470–2472. doi: 10.1093/bioinformatics/btaa997 33258917

[B66] Ye C. Zhou Q. Hong Y. Li Q. Q. (2019a). Role of alternative polyadenylation dynamics in acute myeloid leukaemia at single-cell resolution. RNA Biol. 16, 785–797. doi: 10.1080/15476286.2019.1586139 30810468 PMC6546370

[B67] Ye C. Zhou Q. Wu X. Yu C. Ji G. Saban D. R.. (2019b). scDAPA: detection and visualization of dynamic alternative polyadenylation from single cell RNA-seq data. Bioinformatics 36, 1262–1264. doi: 10.1093/bioinformatics/btz701 PMC8215916 31557285

[B68] Yu Z. Hong L. Li Q. Q. (2022). Signatures of mRNA alternative polyadenylation in arabidopsis leaf development. Front. Genet. 13. doi: 10.3389/fgene.2022.863253 PMC9086830 35559042

[B69] Zhang T.-Q. Xu Z.-G. Shang G.-D. Wang J.-W. (2019). A single-cell RNA sequencing profiles the developmental landscape of arabidopsis root. Mol. Plant 12, 648–660. doi: 10.1016/j.molp.2019.04.004 31004836

[B70] Zhao X. Wu S. Fang N. Sun X. Fan J. (2020). Evaluation of single-cell classifiers for single-cell RNA sequencing data sets. Briefings Bioinf. 21, 1581–1595. doi: 10.1093/bib/bbz096 PMC7947964 31675098

[B71] Zheng G. X. Y. Terry J. M. Belgrader P. Ryvkin P. Bent Z. W. Wilson R.. (2017). Massively parallel digital transcriptional profiling of single cells. Nat. Commun. 8. doi: 10.1038/ncomms14049 PMC5241818 28091601

[B72] Zhou Q. Fu H. Yang D. Ye C. Zhu S. Lin J.. (2019). Differential alternative polyadenylation contributes to the developmental divergence between two rice subspecies Japonica and Indica. Plant J. 98, 260–276. doi: 10.1111/tpj.14209 30570805

[B73] Zhu S. Ye W. Ye L. Fu H. Ye C. Xiao X.. (2020). PlantAPAdb: a comprehensive database for alternative polyadenylation sites in plants. Plant Physiol. 182, 228–242. doi: 10.1104/pp.19.00943 31767692 PMC6945835

